# Comparative analysis of processing speed impairments in TLE, FLE, and GGE: Theoretical insights and clinical Implications

**DOI:** 10.1016/j.ebr.2024.100722

**Published:** 2024-10-28

**Authors:** Adam Falah, Gavin P. Winston

**Affiliations:** a Centre for Neuroscience Studies, Queen’s University, 18 Stuart St, Kingston, Ontario K7L 3N6, Canada; b Department of Medicine, Queen’s University, 76 Stuart Street, Kingston, ON K7L 2V7, Canada

**Keywords:** Processing speed, Epilepsy, Neuropsychology, Cognition, Slowing, Psychomotor speed

## Abstract

In this narrative review, we explore the differences in processing speed (PS) impairments among three epilepsy conditions; Temporal Lobe Epilepsy (TLE), Frontal Lobe Epilepsy (FLE) and Genetic Generalized Epilepsy (GGE) with a focus on Juvenile Myoclonic Epilepsy (JME). Despite the large body of research focusing on cognition in epilepsy, the intricacies of PS impairments in the epilepsy syndromes have not been fully explored. We investigate the cognitive profiles with focus on PS associated with each of the three conditions, and the neuropsychological methods employed. Furthermore, we evaluate PS in epilepsy within the theoretical frameworks of PS, such as the Relative Consequence Model, the Limited Time Mechanism Model, and the Neural Noise Hypothesis. We find the main challenge of PS research in epilepsy is the inconsistency of assessment methods utilized in different studies. Furthermore, PS impairments are not isolated but rather interconnected to other cognitive domains. Thus, future studies need to standardize PS assessment tools, and incorporate innovative solutions such as technology and neuroimaging techniques to further enhance our understanding of PS impairments in epilepsy.

## Highlights

• PS impairment is common in epilepsy, associated with other cognitive deficits.
• PS deficits are specific to certain syndromes or transdiagnostic across epilepsy.
• A combination of theoretical models may help explain PS deficits.
• It is related to factors including epilepsy duration, treatment and genetics.
• Use of standardized assessment approaches is needed in future research.

## 1 Introduction

Epilepsy, a chronic neurological disorder, affects over 70 million individuals worldwide [1]. Characterized by recurrent unprovoked seizures, this condition is also associated with cognitive and psychiatric comorbidities. Significant morphological and functional changes in the brain contribute to impairments in memory, language, and executive function − skills that are essential for daily life [2], [3], [4], [5], [6].

Different epilepsy syndromes display different cognitive profiles and deficits. Temporal lobe epilepsy (TLE), the most common form of focal epilepsy, is often associated with language and memory impairments [7], [8]. In contrast frontal lobe epilepsy (FLE), the second most common focal epilepsy, exhibits deficits in executive function, attention, and motor coordination [9], [10], [11]. Genetic generalized epilepsy (GGE) includes various subsyndromes such as childhood absence epilepsy (CAE) and juvenile myoclonic epilepsy (JME). JME is a particularly well-characterized subtype of GGE, which can also result in widespread cognitive deficits [12], particularly executive functions [13].

Although there has been substantial research into the cognitive profiles of TLE, FLE, and GGE, the intricacies of processing speed (PS) impairments—a prevalent and consequential cognitive dysfunction in epilepsy—remain poorly understood. Specifically, the interactions between PS and other cognitive domains such as memory and executive function necessitate further exploration [14], [15].

PS is commonly recognized as a foundational cognitive domain that underlies an individual’s ability to process information, make decisions, and interact with their environment. It comprises the sensory phase involving the initial reception of stimuli, the cognitive phase which requires processing of this information and relies on sustained attention, and the motor phase which involves the output or reaction [16]. PS efficiency is dependent on the physiological integrity of brain structures [17], [18], [19] and is known to naturally decelerate with age; however, a pronounced slowing may be indicative of the cognitive deficits associated with diseases such as Alzheimer's and vascular dementia [20].

While numerous studies have investigated cognitive impairments in epilepsy, particularly PS, recent research has increasingly focused on this domain. A recent review by Ferrario and Giovagnoli [51] specifically addressed PS impairments in TLE. However, to the best of our knowledge, this review is the first to comprehensively examine PS impairments across four major epilepsy syndromes, offering a unique comparative perspective.

This work evaluates the current body of knowledge regarding PS impairments in epilepsy. We review existing research on PS impairments in common epilepsy syndromes, specifically TLE, FLE, and GGE, including a particular focus on JME, to understand how these impairments manifest differently across syndromes and to evaluate the assessment methods. Moreover, we explore the applicability of three theoretical models – the Relative Consequence Model, the Limited Time Mechanism Model, and the Neural Noise Hypothesis – and hypothesize which model best explains PS deficits in epilepsy.

### 1.1 Theoretical Framework

Understanding the cognitive impairments associated with epilepsy, particularly PS deficits, requires an examination of different theoretical models. We first introduce three models that hypothesize the role and impact of PS impairments in cognitive functioning.

The *Relative Consequence Model* suggests that deficits in PS are foundational, triggering subsequent dysfunction across various cognitive domains such as working memory. Within this framework, PS impairments are not an isolated phenomenon but a primary deficit that cascades into a broader cognitive disruption [21].

The *Limited Time Mechanism Model* suggests that slower processing at the early stages of cognitive operations could compromise the performance of subsequent tasks. This model emphasizes time-based constraints in cognitive processing, where initial delays may have a domino effect, impacting later stages of cognition [22].

The *Neural Noise Hypothesis* addresses the irregular neural activity characteristic of epilepsy. It hypothesizes that seizures could reflect fluctuations in the signal-to-noise ratio of the central nervous system, which could impact cognitive outcomes, including PS. This hypothesis focuses on the neurobiological underpinnings of cognitive impairments in epilepsy [23].

Each of these models offers a different perspective on the potential mechanisms underlying PS impairments in epilepsy. This review applies these theoretical frameworks to synthesize findings from the literature, aiming to identify the most empirically supported model in the context of epilepsy.

## 2 Methods

### 2.1 Search Strategy

A comprehensive literature search was conducted to collect relevant articles in both PubMed and Scopus databases up to and including December 2023. Our search utilized a combination of MeSH terms and keywords, targeting three categories (Table 1): epilepsy-related terms (e.g.,“Epilepsy, Temporal Lobe”, “Myoclonic Epilepsy, Juvenile”, “Epilepsy, Frontal Lobe”, “Epilepsy, generalized”), cognition-related terms (e.g., “*psycholog*”,”cognit*”, “mental”, “process*”, “psychomotor”), and speed of cognition-related terms (e.g., “speed”, “slowing”, “reaction time”).

**Table 1:** Keywords and search strategy.

Database	Search strategy and keywords	
Pubmed	Title and abstract	((“Neuropsychological Tests”[Mesh]) AND (“Epilepsy, Temporal Lobe” OR “Myoclonic Epilepsy, Juvenile” OR “Epilepsy, Frontal Lobe” OR “Epilepsy, generalized” OR “Epilepsy, generalised”) AND (“cognit*” OR “mental” OR “process*” OR “psychomotor”) AND (“speed” OR “slowing”)) OR ((“Neuropsychological Tests”[Mesh]) AND (“Epilepsy, Temporal Lobe” OR “Myoclonic Epilepsy, Juvenile” OR “Epilepsy, Frontal Lobe” OR “Epilepsy, generalized” OR “Epilepsy, generalised”) AND (“Reaction Time”[Mesh]))
SCOPUS	Title and abstract	((“neuropsyc*”) AND (”Epilepsy, Temporal Lobe“ OR ”Myoclonic Epilepsy, Juvenile“ OR ”Epilepsy, Frontal Lobe“ OR ”Epilepsy, generalized“ OR ”Epilepsy, generalised“) AND (”cognit*“ OR ”mental“ OR ”process*“ OR ”psychomotor“) AND (”speed“ OR ”slowing“)) OR ((”neuropsyc*”) AND (“Epilepsy, Temporal Lobe” OR “Myoclonic Epilepsy, Juvenile” OR “Epilepsy, Frontal Lobe” OR “Epilepsy, generalized” OR “Epilepsy, generalised”) AND (“Reaction Time”))

Search results underwent a two-step evaluation process to ensure research relevancy. The initial step involved screening titles, keywords, and abstracts of all the search results. This was followed by full-text analysis to ensure articles meet inclusion criteria. Additionally, a secondary search was performed by examining the references of selected articles to identify additional research papers that may have not been captured in the initial database search. Search results management and duplicate removal were conducted using the EndNote reference manager.

### 2.2 Study Selection

Specific eligibility criteria were:

• Inclusion criteria: Original research papers, published in English with one or more cognitive measures discussing PS, participants with TLE, FLE, or GGE, and a minimum age of 15 years old (as PS matures in the middle of adolescence [52]).
• Exclusion criteria: Review papers, including literature reviews, studies with participants who have additional medical conditions known to affect cognition other than epilepsy (e.g., neurodegenerative diseases). Studies examining post-operative cognitive changes, including those involving patients who have already undergone surgery, and studies missing cognitive measures relating to PS.

## 3 Results

**Fig. 1:**
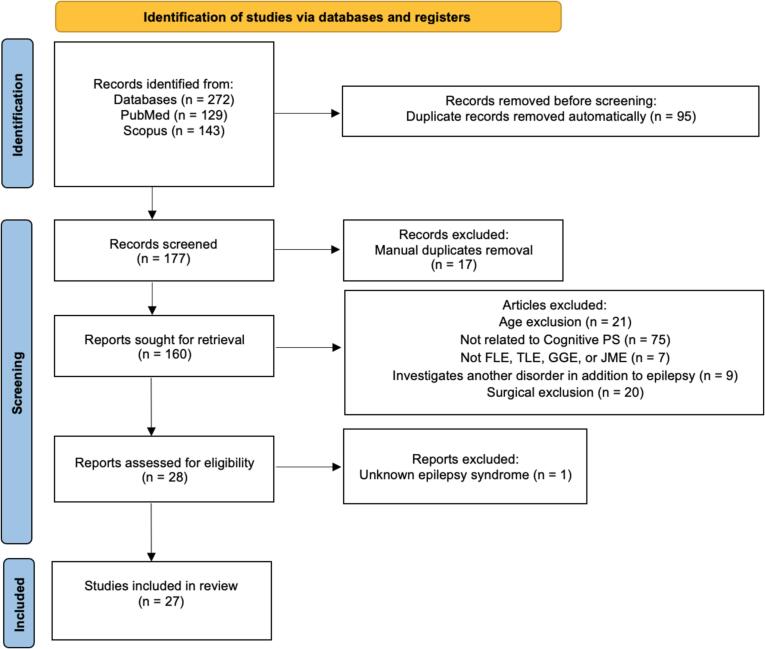
PRISMA flow chart, the process of study selection.

The literature search yielded 272 articles (Fig. 1) from PubMed (n = 129) and Scopus (n = 143) databases. After removing 95 duplicates using Endnote, 177 papers remained. Seventeen more duplicates were manually identified and removed, resulting in 160 articles. Following our exclusion criteria, 133 articles were removed (age exclusion (n = 21), not related to cognitive PS (n = 75), different epilepsy syndromes than our focus (n = 7), presence of another neurological disorder (n = 9), focus on post-surgical outcomes (n = 20), unknown epilepsy syndrome (n = 1)), leaving us with 27 articles.

### 3.1 TLE

Eight studies investigated PS impairments in TLE using various neuropsychological assessments (Table 2). Trail Making Test Part A (TMT-A) was the most common assessment utilized in six studies [24], [25], [26], [27], [28], [29], followed by the Stroop Color and Word Test (SCWT) in three studies [24], [27], [28], and the Symbol Search (SS) from the Wechsler Adult Intelligence Scale − Third Edition (WAIS-III) in two [25], [30].

**Table 2:** Studies focused on processing speed in patients with TLE.

**Reference**	**Purpose**	**Standardized Population Details**	**Assessment Methods**	**Cognitive Domain**	**Key Findings**
[24]	Investigate amygdala volumes vs. cognitive ability in left lateralized TLE, with/without HS	LTLE-HS (n = 14), LTLE-MRIneg (n = 15), controls (n = 30)	Attention Matrices, TMT-A, Stroop Color/Word Interference Test (SCWT)	Attention and PS	No cognitive difference between TLE-HS and TLE-MRIneg groups (p = 0.127), increased left amygdala volume correlated with reduced attention and processing speed (R^2^ = 0.441 to 0.476)
[27]	Describe the development of IC-CoDE and assess its application to multi-centre sample of TLE patients.	N = 2485, Age: 38 years, Education: 14 years	TMT-A, Digit Span (DS), WMS-III Spatial Span, Arithmetic, D-KEFS Color-Word Interference, Stroop Naming/Reading, Coding, SS (WAIS-IV)	Attention and PS	Significant visuomotor sequencing impairment on TMT-A (22 %-35 %); impairments across multiple assessments, including Stroop Reading, Coding, Symbol Search, Digit Span, Spatial Span, and Arithmetic (13 %-30 % range); deficits in attention and processing speed present in 87 % of the Generalized impairment phenotype.
[28]	Evaluate the relationship between slowed PS and other cognitive domains in TLE patients	TLE: N = 100, Age: 36.8 Controls: N = 82, Age: 33.6	Stroop Test (Color Naming), TMT-A, Grooved pegboard test (GPB) (D/N-D hand)	PS & psychomotor speed	TLE showed slower PS and psychomotor speed than controls across tests (p < 0.01). In controls, verbal IQ directly enhanced cognitive functions; in TLE, verbal IQ improvements correlated with better PS and subsequently improved cognitive performance across multiple domains.
[26]	Examine the cognitive profile of PNES patients and a corresponding age-matched group of TLE patients during VEMU admission and one year following their release	PNES (N = 24, Age = 36.6, ASM = 1.4), TLE (N = 24, Age = 37.2, ASM = 1.8)	Rey Auditory Verbal Learning Test (RVALT) Trial 1, Digit Span forward (DSF) (WAIS-III), TMT-A, GPT (DH)	Attention and psychomotor speed	No initial cognitive differences between PNES and TLE (p = 0.33). Attention and psychomotor speed improved in PNES at 1-year follow-up (p = 0.021) but remained the same in TLE. Cognitive improvement in PNES was associated with reduced ASM (anti-seizure medications) use; this was not found in TLE.
[25]	Assess the relationship between neighbourhood deprivation and neuropsychological function in TLE patients	N = 800, Age median = 38.3, Gender = 58 % female	Coding (WAIS-III), symbol search (SS) (WAIS-III), TMT-A	PS	Steady decline in PS with increasing neighbourhood disadvantage (P < 0.001), ranging from a mean score of 99 in the least to 90 in the most disadvantaged areas. [referenced against standard scores with a mean of 100 (SD = 15)]
[29]	Investigate the impact of abnormal physiological arousal and other disease-related factors on neuropsychological deficits in FLE and mTLE patients	FLE (n = 24, Age = 32.45,), mTLE (n = 19, Age = 33.07); majority on polytherapy: FLE (66.67 %), mTLE (84.21 %). Controls (n = 26, matched to patients by gender, age, general intelligence, and education)	Digit symbol coding (DSC) (WAIS-III), TMT-A, Stroop Neuropsychological Screening Test (SNST), Test of Variables of Attention, reaction time (RT), commission error (Com) (TOVA)-V RT, TOVA-V Com	PS & visuomotor speed; concentration, visual scanning, and motor speed; attention and inhibitory control	FLE and mTLE showed statistically significant lower PS compared to controls in all assessments, except in TMT-A (lower performance but not statistically significant). Early disease onset correlated with faster reaction times on TOVA-V RT (p = 0.043); longer disease duration correlated with slower processing speeds on TOVA-V RT (p = 0.029). No significant impact of ASM type on DSC and TOVA-V RT (p > 0.05).
[30]	Assess the relationship between the asymmetry of thalamic subregions and cognitive performance in TLE patients using morphological and metabolic imaging data	Population details: L-TLE (N = 24, median age = 35.3), R-TLE (N = 15, median age = 40)	DSC (WAIS-III), SS (WAIS-III)	PS	Asymmetrical thalamic metabolism correlates with PS index (PSI). In L-TLE: higher right thalamic metabolism correlated with improved PSI in lateral, intralaminar, and medial thalamic subregions (p = 0.002, 0.002, 0.019, respectively), and the entire thalamus (p = 0.01). R-TLE: improved PSI correlated with larger left anterior subnuclei volume (p = 0.006); higher metabolic activity in the left medial subnucleus and entire left thalamus (p = 0.024, p = 0.012, respectively) correlated with better PSI.
[31]	Investigate oculomotor performance in drug-resistant epilepsy patients (DRE)	Patients with focal drug-resistant epilepsy (DRE): N = 51, Age = 37.4. Controls: N = 31, Age = 33.6	Computerized ocular test (COT)	Cognitive Processing Speed	Slower PS with longer antisaccade (AS) latency in TLE compared to controls (mean difference of 47.6 ms (p = 0.005)). Reduced motor coordination and inhibitory control in TLE and FLE; FLE most affected, showing a significantly higher mean AS error rate compared to controls (FLE: 34.5 %, controls: 18.8 %, p = 0.007; TLE: 31.5 %, controls: 18.8 %, p = 0.016)

Patients with TLE consistently exhibited impaired PS compared to healthy controls across several tests (p < 0.01) [28] with drug-resistant TLE subgroup showing even greater slowing compared to controls [31]. Impairments in attention and PS were modality-independent as deficits were observed across different test formats—timed and untimed, auditory and visual, and with and without motor components [27]. Furthermore, longitudinal data indicated no significant change in attention and psychomotor speed over one year [26].

One study highlighted the impact of anatomical factors on PS impairment, specifically linking increased left amygdala volume to reduced attention and PS [24]. Another study investigated the influence of environmental factors affect PS and found that PS scores dropped by 0.6 SD from the least to the most disadvantaged neighbourhoods [25].

### 3.2 FLE

Four studies examined PS impairments in FLE (Table 3), with TMT-A being the most used assessment method [9], [32], [33], [34].

**Table 3:** Studies focused on patients with FLE.

Reference	Purpose	Standardized Population Details	Assessment Methods	Cognitive Domain	Key Findings
[32]	Apply the IC-CoDE to patients FLE using multicenter data	N = 455, Age: 33 years	TMT-A, SS, Coding	PS	Impairments in multiple domains. TMT-A showed impairment rates of 34 %-50 %. Unique cognitive profile of FLE with significantly higher rates of attention and PS impairments compared to TLE.
[9]	Identify distinct cognitive phenotypes in FLE and investigate the causes of its cognitive differences	N = 106, Age = 33.99	Coding (WAIS) , SS (WAIS), TMT-A	PS	Four cognitive phenotypes identified include 25 % Generalized (most severe PS impairments). Significant PS differences across cognitive phenotypes: Generalized vs. Domain-Specific and Intact phenotypes (p < 0.001), and Tri-Domain vs. Intact (p = 0.003)
[34]	Investigate cognitive impairments linked to FLE to provide localization information for the preoperative evaluation for epilepsy surgery candidates	FLE: N = 34, Age = 33.26, Education: 11.76, MTLE: N = 37, Age: 31.92, Education: 11.38. controls: N = 22, Age: 33.09, Education: 12.50	TMT-A	PS, visual scanning, concentration, motor function	Similar results on TMT-A in FLE, mTLE, and controls (p > 0.05). TMT performance not affected by lesion presence or lateralization in FLE. TMT not affected by epilepsy duration and seizure frequency.
[33]	Examine visuomotor set-shifting deficits in FLE compared to those with TLE and HC	FLE: N = 23, Age = 36.8, Education = 13.8. TLE: N = 20, Age = 37.9, Education = 13.5. Controls: N = 23, Age = 36.9, Education: 13.8	D-KEFS TMT (visual scanning, number sequencing, letter sequencing, number-letter switching, motor speed)	psychomotor speed & set-shifting	Reduced number-letter switching in FLE vs TLE (p < 0.05) and Controls (p < 0.01). No difference in set-shifting in TLE vs Controls (p > 0.05). More set-loss errors in FLE compared to TLE and controls (p < 0.01). Less education linked to longer task completion time (r = -0.425, p < 0.05)

Patients with FLE showed greater PS impairments compared to TLE across multiple domains [32]. They also had reduced performance on number-letter switching task compared to TLE and controls [33]. One study revealed distinct cognitive phenotypes in FLE patients with varying degrees of PS impairments [9]. However, another study identified similar performance on TMT-A across FLE, mesial temporal lobe epilepsy (mTLE), and controls, with no significant differences observed [34].

### 3.3 GGE

Five studies investigated revealing PS deficits and selective vulnerabilities in different PS aspects (Table 4). Coding was the most commonly used assessment among the various neuropsychological tests [35], [36].

**Table 4:** Studies focused on patients with GGE.

Reference	Purpose	Standardized Population Details	Assessment Methods	Cognitive Domain	Key Findings
[35]	Describe the neuropsychological profile of a group of patients with different IGE syndromes with simultaneous video electroencephalography (EEG)	JME: N = 19, Age = 33, VPA Dose Median = 950 mg. CAE/JAE: N = 20, Age = 34.4, VPA Dose Median = 1550 mg. IGE-GTCS: N = 22, Age = 29.9, VPA Dose Median = 1000 mg.	WAIS-III: coding, Stroop, Rey Figure Copy Time (RFTC)	PS	Reduced PS in patients compared to controls with significant difference observed in Stroop words test (p = 0.019), Coding test (p = 0.045), Rey Figure-time (p = 0.046). Specific impact of VPA on PS and working memory, but not on overall cognitive function.
[38]	Investigate the relationship between epileptic seizures, memory performance and neuronal dysfunction in the temporal lobes of a group of patients with IGE.	N = 29, Age = 30. Controls: N = 15, Age = 34.	SCOLP (Spot-the-Word, Speed of Comprehension)	Information processing speed (IPS)	Patients underperformed compared to controls on SCOLP, in both Speed of Comprehension (patients: mean = 53, controls: mean = 67, p = 0.031) and Spot the Word sections (patients: mean = 46, controls: mean = 51, p = 0.020)
[39]	Investigate the link between neuropsychological characteristics with the endophenotype of epilepsy and treatment response in Idiopathic Generalized Epilepsy (IGE)	Controlled Seizures: N = 72, Age = 36.2. IGE Subsyndrome distribution: JME 50 %, JAE 22.2 %, GTCA 27.8 %. Average ASM tried: 2.5. Uncontrolled Seizures: N = 34, Age = 32.5. IGE Subsyndrome distribution: JME 44.1 %, JAE 35.3 %, GTCA 20.6 %. Average ASM tried: 2.9	Purdue Pegboard test (PPT)	Psychomotor speed	Significant deficits in psychomotor speed in IGE patients compared to norms as revealed by their Purdue Pegboard test scores (p < 0.0001). Consistent psychomotor deficits across GGE subtypes and is not affected by the seizure control status or treatment.
[37]	Investigate the characterization of cognitive function of GGE	N = 76, Age = 29, Syndrome: CAE 13 %, JAE 28 %, JME 26 %, GTCSO 30 %, Another 3 %	Woodcock-Johnson III: test 6, 20	IPS: decision speed, visual matching	GGE showed significantly lower scores on speed of information processing compared to the normative group, with a mean score of 91.21 (SD = 14.46) versus a norm of 100 (SD = 15)
[36]	Investigate the pathophysiology of JME and IGE-GTCS-only related to neuropsychological dysfunction	N = 26, Age: 27.04, Type of IGE: JME 15 patients, GTCS 11 patients. Controls: N = 26 demographically matched	TOVA-A RT, TOVA-V RT, DSC, TMT-A	PS, visual scanning, concentration, psychomotor function	IGE patients had significantly slower reaction times than controls in PS tasks: Visual TOVA-RT (p = 0.003) and Auditory TOVA-RT (p = 0.003), indicating impairments in both visual and auditory PS. No significant differences between IGE patients and controls in TMT-A, and DSC.

Loughman, Bowden and D'Souza [37] identified gaps in speed of information processing relative to normative data. CAE/JAE and JME subtypes exhibited PS deficits compared to controls in the Stroop words and Coding tests with patients treated with VPA performed worse than those not taking it [35].

Deficits in comprehension speed, word recognition and psychomotor speed were observed in GGE [38], [39]. Notably, reduced psychomotor speed was consistent across GGE subtypes and unaffected by seizure control status [39]. Delays in reaction times in visual and auditory PS tasks were observed, while other aspects of PS remained intact, including visuomotor coordination (assessed via DSC) [36].

### 3.4 JME

Ten studies examined PS in JME (Table 5) with TMT-A being the most used neuropsychological assessment [40], [41], [42], [43], [44], [45], [46], [47].

**Table 5:** Studies focused on patients with JME.

Reference	Purpose	Participants Details	Assessment Methods	Cognitive Domain	Key Findings
[45]	Explore the cognitive performance in newly diagnosed JME with a focus on executive function and verbal memory	JME: N = 18, Age: 18.1. Education: 10.5, Before ASM: 11 patients; After ASM: 5 patients with Monotherapy, 2 patients with Polytherapy. Control: N = 18, Age: 18.7, Education: 10.7	WAIS Digit Symbol, Alternating S task, finger tapping, TMT, and Stroop test	PS and executive function, psychomotor speed	No significant differences in PS between JME patients and controls; Alternating S (p = 0.568), Digit Symbol (p = 0.192), and Tapping (p = 0.488). No difference in psychomotor speed in JME before or after ASM treatment compared to controls; Alternating S (p = 0.724), Digit Symbol (p = 0.092), Tapping (p = 0.726)
[46]	Investigate perception and recognition of emotions in JME via fMRI and neuropsychological tests	Controls: N = 68, Median age: 24 years, Median education: 12 years. JME: N = 65, Median age: 27 years, Median education: 12 years	TMT-(A & B) along with the Test of Attentional Performance (TAP)	Psychomotor speed, mental flexibility, rection time, inhibition	Slower psychomotor speed in JME vs controls on TMT(median time: 28.50 s vs 22.00 s, p < 0.001). Diminished mental flexibility in JME vs controls on TMT-B (median time: 60.00 s vs 48.00 s p = 0.016). Slower reaction times and inhibition in JME vs controls on the TAP GoNogo test (median reaction time:0.413 s vs 0.379sp = 0.001)
[47]	Explore whether JME is associated with higher artistic creativity due to disinhibited frontal lobe function (paradoxical functional facilitation (PFF))	JME: N = 25, Age: 29 years TLE: N = 25, Age: 30.3 years Controls: N = 25, Age: 28.2 years	TMT-A, TMT-B	psychomotor speed, mental flexibility	Slower psychomotor speed and mental flexibility in JME compared to TLE and controls on TMT-B (mean time: 67.6 s vs 54.6 s vs 52.8 s respectively). Although did not meet stricter significance criteria (p = 0.038)
[48]	Assess cognition including intellectual function, memory, language and naming, executive function, and the impacts of epilepsy and ASMs side effects in JME patients.	N = 60, Age (median) = 31 Education (median) = 13	WAIS III: PSI; (DSC, SS)	PSI	Patients got a lower PSI score, a mean of 86.0, compared to the norm median of 100 (SD = 15) (p < 0.001). Lowest scores in DSC with a mean of 7.0, below the norm of 10 (SD = 3) (p < 0.001). Lower SS scores, with a mean of 8.3, below the norm mean of 10 (SD = 3) (p < 0.001).
[44]	Compare neuropsychological function in JME and FLE	JME: N = 16, Age: 27.31, Education: 13.62, Patients on Polytherapy: 62.5 %. FLE: N = 34, Age: 33.26, Education: 11.76, Patients on Polytherapy: 70.5 %. Controls: N = 48, Age: 29.812, Education: 13.05	TOVA, DSC WAIS-II subtest, TMT-A	Psychomotor speed, visuomotor coordination	Slower PS in FLE vs JME and controls; FLE mean reaction times on TOVA Visual RT (775.92 ms), JME (490.19 ms), controls (430.92 ms). Slower PS in FLE vs JME and controls; on DSC FLE mean score of 14.67, JME (66.44), controls (43.58). Reduced PS for FLE on TMT-A, mean completion times 47.15 s, JME (38.44 s) and controls (36.79 s)
[43]	Assess cognitive impairment in JME and its link with epilepsy-related factors and education	Participants Details: JME: N = 50, Age: 26.2, Years of Education = 10.8 Control: N = 50, Age: 26.3, Years of Education = 10.9	WAIS III subtests including DS and SS the Stroop Test, and TMT	psychomotor speed, visual processing speed, attention, mental flexibility, control of inhibition, and cognitive speed	Significant deficits in PS and psychomotor measures in JME compared to controls with z-scores significantly lower in the DS (−0.431, p = 0.003), SS (−0.575, p = 0.001), TMT-A (−1.760, p < 0.001), TMT-B (−2.098, p < 0.001), Stroop Test (Color: −0.867, Word: −0.695 with p = 0.019, Interference: −1.346, all p < 0.001)
[49]	Explore cognitive functioning in JME patients and their siblings under video-EEG	JME: N = 22, Age = 26.7, Education = 14.6. JME unaffected Siblings: N = 22, Age = 26.6, Education = 15. Controls: N = 44, Age = 25.7, Education = 14.6	GPB(DH) and Stroop Test	Psychomotor speed	No difference among groups in Stroop Test. Slower psychomotor function in JME and unaffected siblings vs controls as shown by GPB (DH); similar completion times in JME (82.8 s) and unaffected siblings (82.7 s) vs to controls (75.9 s) p = 0.05
[42]	Examine social cognition, and its link to executive function in JME patients	JME: N = 20, Age = 26.7, Education: 14.6, Duration = 12.7. Controls: N = 20, Age: 26.2, Education: 15.2	TMT-A and TMT-B and the Stroop Color-Word Test	Psychomotor speed and shifting abilities, selective attention and inhibition	Longer TMT-A completion times for JME (50.6 s) vs controls (41.4 s), p = 0.044. No difference in TMT-B, Stroop Color-Word Test measures (time interference, error rate)
[40]	Investigate whether JME is associated with changes in the dopamine system and if such changes are linked to their interictal dysfunctions	JME: N = 12, Age = 38, Education = 13, Duration: 23. Controls: N = 12, Age: 36, Education = 13	TMT-(A & B), finger tapping, GPB, Dual Task Condition Test, and DS	Psychomotor speed, executive function, working memory reaction time	Deficits in psychomotor speed, executive functions, and working memory reaction times in JME vs controls [p-values 0.003–0.042]
[41]	Compare neuropsychiatric profiles of JME patients on VPA or TPM monotherapy to explore links between cognitive dysfunction, psychiatric disorders, and epilepsy-related factors	Patients treated with VPA: N = 26, Age: 24.8, Epilepsy duration (mean): 11.3, Dosage: 992.8 mg/day (range 500–1750) Patients treated with TPM: N = 16, Age: 22.1, Epilepsy duration (mean): 9.8, Dosage: 98.3 mg/day (range 50–175)	WAIS-III: SS, Digit Symbol. TMT-A, TMB Stroop I, II, III	PS, attention, mental flexibility	Lower scores in TPM group on SS compared to VPA group (p < 0.05). No difference in DS between groups. Lower scores in TPM compared to VPA group on (Stroop tests, TMT-A, TMB); differences did not reach significance

Across these studies, patients with JME showed deficits in different constructs of PS. Specifically, psychomotor speed and mental flexibility [46], [47], [48], attention shifting and visuomotor coordination [44], and more complex cognitive abilities [40], [41], [42], [43].

Furthermore, unaffected siblings of patients with JME showed reduced psychomotor speed versus controls suggesting genetic predisposition [49]. However, people with a new diagnosis of JME showed PS comparable to that of controls before and after ASM treatment [45].

### 3.5 PS impairments across epilepsy Subtypes

Our review of PS impairments in TLE, FLE, GGE, and JME indicates TMT is the most utilized assessment tool, highlighting its effectiveness in evaluating PS. This investigation reveals a complex picture of PS impairments in epilepsy with some studies showing comparable PS in patients and controls, while others showed significant differences. External factors including ASMs, genetic predisposition, and neighborhood disadvantage further influence PS. Notably, GGE and TLE exhibited a broad range of PS deficits, while FLE and JME showed more selective ones.

## 4 Discussion

### 4.1 Approach to assessment

Impaired PS is prevalent among all examined epilepsy types (TLE, FLE, GGE, and JME), but researchers employ different neuropsychological assessments to evaluate the degree of these impairments. TMT, an established neuropsychological instrument for assessing attention, PS, and executive function, is the most used assessment method across the different types of epilepsy. Other assessments including Stroop Naming/Reading, Coding, and Symbol search have also been employed, in addition to others discussed in the results section. Nevertheless, there is often a lack of clearly disclosed rationale by authors for their assessment choice. This absence of a systematic approach in selecting neuropsychological instruments undermines the comparability of research findings and thwarts the advancement of a consistent understanding of PS impairments in epilepsy.

TMT particularly TMT-A, and WAIS subtests including Coding and Symbol Search are more prevalent tools for TLE and FLE research. However, researchers use similar neuropsychological methods differently. For instance, some authors refer to TMT-A, Attention Matrices, and Stroop Color/Word Interference to measure attention and PS [24], while others used TMT-A, RAVLT, and Digit Span forward subtest of the WAIS-III and the Grooved Pegboard dominant hand score as measures of attention and psychomotor speed (defined as the speed of the motor response to a stimulus) [26]. Both authors used TMT-A although they used it with other neuropsychological tests to measure similar cognitive domains. Such discrepancies are common in the literature, which leads to inconsistent interpretations of results.

Additionally, many studies suggest that these assessments measure interconnected cognitive domains, with PS and attention commonly paired in what is assessed [24], [26]. This overlap indicates that observed deficits may reflect impairments in both domains rather than PS alone, requiring careful interpretation of findings. Moreover, this highlights the need for a more systematic approach to assessment selection to ensure that the intended cognitive domain is accurately captured. Future research should either select assessments that better isolate these domains or explicitly account for this overlap in their analyses to enhance the precision of their conclusions.

### 4.2 Association with broader cognitive functions

PS is a critical cognitive function that utilizes widespread brain areas and integrates with broader cognitive functions in epilepsy. For instance, in TLE, higher verbal intelligence was associated with better PS, positively affecting executive function, memory, and working memory. This association was particularly absent in the control group [28]. Similarly, PS and cerebral white matter volume throughout the brain are correlated in TLE [50].

Similar associations were observed in other types of epilepsy. For example, in FLE, reductions in PS and attention reductions appeared to be the main cognitive domains distinguishing Tri-Domain Phenotype (impairment in three domains) from the Domain-Specific Phenotype (impairment in one or two domains). The authors suggest PS impairment in FLE may be linked to broader neural networks, highlighting the involvement of fronto-subcortical networks. They recommend exploring patterns of white matter integrity within these networks for deeper insights into PS in FLE [9].

In GGE, reductions in overall cognitive ability, acquired knowledge, and PS suggest a pathway where deficits in PS exacerbate memory difficulties, thus impairing the retention of learned information and reflecting reduced acquired knowledge. This hypothesis underscores the potential cumulative effects of PS deficits on broader cognitive functions [37].

### 4.3 Theoretical model implications

Synthesizing the literature on PS impairments in epilepsy unveils an integrated hypothesis that navigates the complexities of PS deficits, combining elements from the Relative Consequence Model, the Limited Time Mechanism Model, and the Neural Noise Hypothesis. Such an approach explains the multifaceted nature of PS impairments and aligns with the insights derived from studies within the field.

Studies have underscored PS deficits as foundational to broader cognitive impairments, aligning with the Relative Consequence Model. For instance, disparities in cognitive functions, including executive function, and working memory, are mediated by PS, particularly in TLE not in controls [28]. Similarly, a significant majority within the Generalized impairment phenotype population exhibits deficits in attention and PS, pointing to the widespread effects of these deficits [27].

The Limited Time Mechanism Model demonstrated through findings emphasizing the cascading effect of initial processing delays on subsequent cognitive performance. FLE patients, for example, show impairments not only in initial sensory and cognitive processing as measured through (TOVA Visual RT), but also in more complex cognitive and motor functions (TMT-A and DSC (WAIS-III)) which illustrates a gradient of deficits [44]. In JME, delays across a range of tests further elucidate the breadth of PS impairment, from basic psychomotor speed to tasks requiring advanced cognitive integration [46].

Support for the Neural Noise Hypothesis arises from studies identifying neurobiological disruptions as foundational for PS impairments. Patients with TLE, for instance, exhibit correlations between anterior subnuclei volume and thalamic metabolism with PSI (processing speed index) [30]. The involvement of the thalamus, as a critical hub in cognitive processing networks, underscores its significant role in the cognitive deficits observed in TLE.

Additionally, structural changes in the brain, such as increased amygdala volume, have been linked to attention and PS deficits [24]. The amygdala is traditionally associated with emotional processing, but it interacts with other brain areas that influence cognitive functions, including attention and PS. Emotional states regulated by the amygdala can further affect cognitive processing, potentially exacerbating PS impairments in TLE.

Oculomotor performance has also been linked to cognitive dysfunction in TLE [31]. Oculomotor tasks are sensitive to deficits in PS. Therefore, such finding provides further evidence of the complex cognitive impairments associated with this syndrome, reflecting both sensory and motor processing challenges.

This comprehensive examination reveals that no single theoretical framework suffices to fully explain PS deficits in epilepsy. Instead, adopting a multifaceted lens incorporating foundational PS impairments, timing of cognitive processing, and neurobiological influences provides a more complete understanding.

### 4.4 Role of epilepsy characteristics and treatment on PS

PS impairments in epilepsy are influenced by factors beyond the direct impact of seizure activity and the use of ASMs. Studies indicate that reduced PS is significantly correlated with the duration of epilepsy and the number of ASMs used in TLE and FLE [28], [29]. In the case of GGE, valproate (VPA) intake is associated with worse performance in PS and visual working memory tests but not the other tests [35]. Furthermore, in JME, patients treated with topiramate (TPM) monotherapy, performed worse than those treated with VPA monotherapy on tasks of attention, short-term memory, PS, and verbal fluency [41]. Besides these clinical features, PS impairments are also impacted by genetic predisposition. Specifically, patients with JME and their unaffected siblings both exhibited reduced psychomotor speed compared to controls [49].

TLE is characterized by pronounced PS deficits that often cascade into broader cognitive impairments. These impairments particularly affect memory and language processing due to the temporal lobe's critical role in these functions. In contrast, FLE predominantly impacts executive functions and motor coordination, as the frontal lobe is integral to these domains. In FLE, these deficits manifest as impairments in both cognitive processing speed and motor response times.

GGE, including JME, presents more diffuse PS impairments, likely due to the generalized nature of seizures and widespread neural disruption involved. In JME, these impairments are particularly apparent in tasks requiring psychomotor speed and coordination, reflecting the generalized and often bilateral nature of the underlying neural disturbances.

Together, these findings from studies on TLE, FLE, and GGE suggest that a multitude of factors, including syndrome type, disease duration, treatment specifics, and genetic predisposition, are fundamental in the manifestation of PS impairments. These insights highlight the importance of incorporating cognitive assessments tailored to the specific challenges associated with each epilepsy syndrome. Additionally, understanding the differences in the impact of anti-seizure medications on PS can guide clinicians in the selection and adjustment of treatments to minimize cognitive side effects. Therefore, personalized approaches that consider the unique clinical profile of each patient with epilepsy are necessary for the assessment and management of their cognitive challenges.

## 5 Conclusion and future Direction

In conclusion, PS impairments are prevalent across the different epilepsy syndromes, and show complex interactions with other cognitive domains. Current research lacks a consistent method to selecting the neuropsychological tools to assess PS. This makes it challenging to compare research findings and reach reliable conclusions.

Moving forward, future studies should aim to establishing a standardized protocol to assist researchers in choosing appropriate assessment tools. Furthermore, incorporating technology into neuropsychological research, represents a promising effort to isolate PS phases (sensory, cognitive, motor) for more detailed evaluations. Additionally, utilizing neuroimaging methods and longitudinal studies could help in better understanding the changes in cognition, and the structural correlates associated with PS.

By adopting these strategies, we can significantly advance our understanding of PS in epilepsy and contribute to more effective treatment strategies for patients.

## CRediT authorship contribution statement

**Adam Falah:** Writing – original draft, Methodology, Investigation, Conceptualization. **Gavin P. Winston:** Writing – review & editing, Supervision, Methodology, Funding acquisition.

## Declaration of competing interest

The authors declare that they have no known competing financial interests or personal relationships that could have appeared to influence the work reported in this paper.
